# Protection against UVB-Induced Wrinkle Formation in SKH-1 Hairless Mice: Efficacy of Tricin Isolated from Enzyme-Treated *Zizania latifolia* Extract

**DOI:** 10.3390/molecules23092254

**Published:** 2018-09-04

**Authors:** Joo-Myung Moon, Se-Ho Park, Kwang-Hwan Jhee, Seun-Ah Yang

**Affiliations:** 1 BTC Corporation, Sangnok.gu, Ansan 15588, Korea; mhjj1919@btcbio.com; 2 Institute of Natural Science, Keimyung University, Daegu 42601, Korea; p86ks1@naver.com; 3 Department of Applied Chemistry, Kumoh National Institute of Technology, Gumi 39177, Korea; khjhee@kumoh.ac.kr; 4 Department of Food Science and Technology, Keimyung University, Daegu 42601, Korea

**Keywords:** MMPs, skin aging, tricin, collagen, *Zizania latifolia*

## Abstract

Tricin, a flavone found mainly in rice bran and sugarcane, has various beneficial effects. It has proven to be a clinically safe and selective potent inhibitor of different cancer cell lines. In this study, we evaluated the efficacy of enzyme-treated *Zizania latifolia* (ETZL) and its major active compound tricin on skin photoaging in SKH-1 hairless mice. Tricin (0.3 mg/kg) and ETZL (50, 150, and 300 mg/kg) were orally administrated to mice for 14 weeks; no cytotoxicity was observed during the entire experimental period. After UVB exposure, we observed significant increases in keratinization, coarse wrinkles, loss of moisture, thickened epidermis, and collagen fiber degradation in the dorsal skin. These features of photoaging were significantly suppressed after oral administration of tricin or ETZL. In addition, the protein expression of collagen effectively increased in ETZL (150 and 300 mg/kg)-treated mice, while the increased metalloproteinase (MMP)-1 and MMP-3 expressions were reduced after exposure to tricin or ETZL, although the effects were not dose-dependent. These data indicate that ETZL may be effective for attenuation of UVB-induced skin damage and photoaging in hairless mice, possibly by inhibiting MMPs expression.

## 1. Introduction

Tricin (4′,5,7-trihydroxy-3′,5′-dimethoxyflavone) identified from various plants is reported to inhibit the growth of colorectal carcinoma [1] and breast cancer cells [2] as well as influenza virus activity [3]. Several studies have demonstrated that tricin isolated from Njavara rice has anti-inflammatory and antioxidative properties [4,5]; tricin and its derivatives isolated from *Zizania latifolia* (ETZL) were previously identified as the active components for inhibition of allergies and inflammation [6]. *Z. latifolia* (Gramineae) is a perennial aquatic plant found in lakes, ponds, and wetlands, and is cultivated in Russia, Korea, Japan, and China [7]. Its culms and rhizomes have long been used to treat kidney, liver, and heart disorders in China, and recent studies have shown that *Z. latifolia* grains have various biological effects that include inhibition of hyperlipidemia, oxidative stress, reducing blood glucose levels, improving insulin resistance, and anti-obesity effects [8]. A small number of studies using the aerial part of *Z. latifolia* revealed inhibitions of H_2_O_2_-induced apoptosis in Neuro2A cells [9], angiotensin-converting enzyme and oxidative stress [10], and antigen-induced allergy in RBL-2H3 cells [11].

Exposure to ultraviolet (UV) radiation leads to skin aging, including wrinkling, sagging, dryness, and erythema reaction. UVB is the most harmful constituent of UV radiation and causes photosensitive reactions and damage at the molecular level, which are largely due to excessive reactive oxygen species (ROS) produced in the epidermis. ROS then induce secretions of metalloproteinase families (MMPs), such as MMP-1, -2, -3, -9, and -13, in skin fibroblasts and keratinocytes, which in turn degrade the collagen and other extracellular matrix (ECM) proteins, impair collagen synthesis, and cause wrinkle formation and skin photoaging [12]. Numerous studies have shown antioxidant-rich natural extracts are capable of blocking UV-stimulated MMP production and secretion in human dermal fibroblasts (HDFs) [13]. In addition, several antioxidant materials have been shown to attenuate the risk of UVB-induced skin damage by topical application in mice [14,15]. Furthermore, recent studies have demonstrated the protective effects of oral administrations of various phenolic compounds from natural herb extracts against photoaging in UVB-exposed hairless mice. It is suggested that the efficacy of oral administration can be increased by combining it with a topical application for anti-photoaging [16]. In a previous study, we found that tricin exerts preventive effects by inhibiting MMPs expression and type-I procollagen degradation via upregulation of antioxidant enzymes, reduced ROS generation, as well as MAPK/AP-1 signaling in UVB-irradiated HDFs (unpublished data). Furthermore, the in vitro data suggest that the extract exerts its efficacy in UVB-induced photoaging in hairless mice through the effect of tricin as an active molecule in ETZL. Nevertheless, there is no report on the anti-wrinkle effect of tricin and the in vivo efficacy of the aerial part of the plant. Thus, the current study investigated the protective efficacy of ETZL and its active compound tricin on histological changes and their effect on expressions of collagen and MMPs.

## 2. Results and Discussion

### 2.1. Estimation of Tricin in ETZL

As demonstrated in our previous study, tricin and its derivatives, including a new flavonolignan salcolin D, were isolated as the active components exerting the anti-inflammatory and anti-allergic activities in ethanol extracts from the aerial parts of *Z. latifolia*. Lee et al. [17] reported the presence of new flavonolignan groups along with tricin in this plant. In our previous study, the quantitative analysis of 11 compounds present in the ethanol extract from the aerial parts of *Z. latifolia* (including tricin and its derivatives) confirmed that tricin is the quantitatively major component in the extract (unpublished data). Additionally, we also confirmed the effectiveness of tricin and the extract to enhance collagen synthesis and to suppress collagen degradation in HDF cells. The present study first investigated the enzyme-treatment conditions such as enzyme type and incubation time, in order to enhance the extraction yield as well as the amount of tricin content in the extract from *Z. latifolia* to promote industrial utilization as a component in inner beauty products. Of the various hydrolases evaluated, the extraction yield with an enzyme mixture (cellulose, hemicellulose, and pectinase) treatment (17.5%) was the highest and resulted in a significant increase as compared to the non-hydrolyzed extract (10.7%) for 24 h incubation at 35 °C. In addition, we determined the effects of varying incubation times with the enzyme (4–24 h) on the extraction yield; treatment with the enzyme mixture for 16 h exhibited the highest yield (17.5%) and tricin content as compared to 24 h incubation (Table 1). Taken together, we propose the optimal conditions of enzyme treatment for ETZL preparation were 0.6% (*w*/*w*) of the enzyme mixture at 35 °C for 16 h, which was used for further experiments in SKH-1 hairless mice. Simultaneously, tricin as a marker molecule was also administrated orally based on the activities data in HDFs and retinoic acid, which is well known for its anti-photo-aging effect in both oral and topical applications, was additionally examined as a topical application in UVB-irradiated mice [18,19].

### 2.2. Effects on Body Weight and Serum Biochemical Indicators

**Figure 1:**
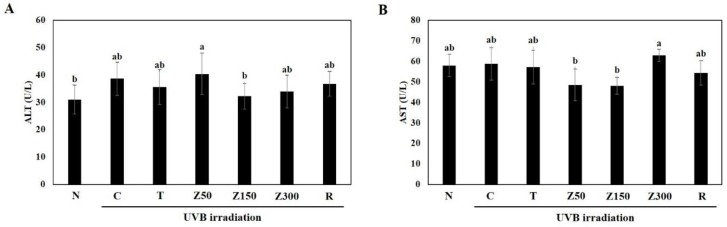
Effects of tricin and ETZL on serum biochemical indicators in UVB-irradiated SKH-1 hairless mice. (**A**) Serum alanine transaminase (ALT) and (**B**) aspartate transaminase (AST) activity in the hairless mice. N, normal control; C, UVB-irradiated control; T, tricin (0.3 mg/kg) oral administration with UVB irradiation; Z50, ETZL (50 mg/kg) oral administration with UVB irradiation; Z150, ETZL (150 mg/kg) oral administration with UVB irradiation; Z300, ETZL (300 mg/kg) oral administration with UVB irradiation; R, UVB irradiation with dermal application of 0.05% retinoic acid. Values are expressed as mean ± SD of nine mice. Different letters show a significant difference at *p* < 0.05 as determined by Duncan’s multiple range test.

In order to examine the toxicity of tricin and ETZL, we evaluated the body weight and biochemical indicators changes, including alanine transaminase (ALT) and aspartate transaminase (AST), in hairless mouse serum. Daily food intake and body weight gain were not significantly different between all of experimental groups during the 14-week experimental period (data not shown), indicating that food intake and body weight change are not influenced by UVB exposure and sample administration. Although tricin and *Z. latifolia* are edible materials, administration of the enzyme-treated extract for a considerably long period in this experiment directed us to determine the hepatotoxicity by measuring ALT and AST concentrations in serum. As shown in Figure 1, there were no significant changes observed in the two liver enzymes among the groups, indicating no hepatotoxicity of tricin or ETZL.

### 2.3. Effects on Wrinkle Formation

Previous physiological studies on *Z. latifolia* and tricin have demonstrated various effects, such as anti-oxidative, anti-inflammation, and anti-allergy effects. However, we believe this is the first in vivo study evaluating the preventive effects of orally administrated tricin or ETZL against UVB-induced skin photoaging. In vitro studies using UVB-irradiated HDFs revealed that the cytotoxicity and ROS overproduction were reduced by exposure to tricin or ETZL, implying protection of the dermal cells against UVB-induced cytotoxicity. Since ROS plays a major role in UVB-induced skin damage and photoaging, anti-oxidative effects of bioactive compounds and extracts from various natural resources on anti-photoaging have been reported [13].

**Figure 2:**
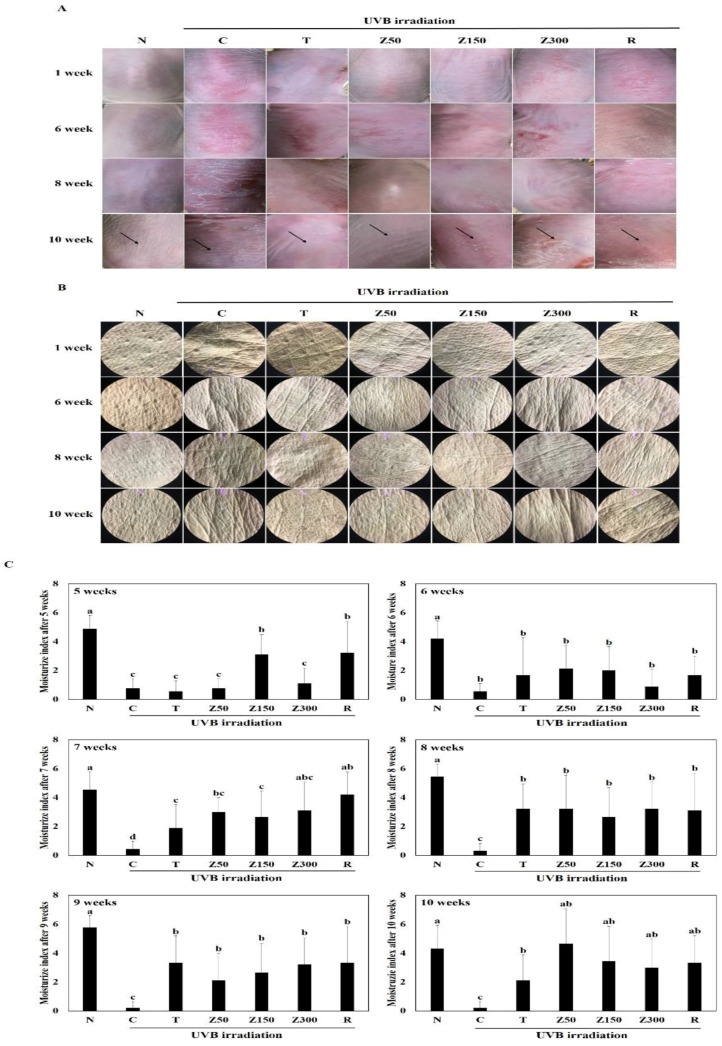
Effects of tricin or ETZL on wrinkle formation in UVB-irradiated SKH-1 hairless mice. (**A**) Microscopic morphological observations at 1, 6, 8, and 10 weeks; (**B**) Images of skin replicas using SILFLO at 1, 6, 8, and 10 weeks; (**C**) Effects on moisture contents at 5 to 10 weeks. Each bar represents the mean ± SD (*n* = 9). N, normal control; C, UVB-irradiated control; T, tricin (0.3 mg/kg) oral administration with UVB irradiation; Z50, ETZL (50 mg/kg) oral administration with UVB irradiation; Z150, ETZL (150 mg/kg) oral administration with UVB irradiation; Z300, ETZL (300 mg/kg) oral administration with UVB irradiation; R, UVB irradiation with dermal application of 0.05% retinoic acid. Values are expressed as mean ± SD of nine mice. Different letters show a significant difference at *p* < 0.05 as determined by Duncan’s multiple range test.

In this study, we investigated the prominent features of skin photoaging in UVB-exposed SKH-1 hairless mice, including the increase of keratinization, erythema reaction, wrinkling, and the loss of dorsal skin moisture. To assess whether tricin or ETZL administration prevents photoaging induced by UVB irradiation, changes in the dorsal skin surface of hairless mice were observed by visual assessment in each group at 1, 6, 8, and 10 weeks of treatment (Figure 2A). We observed that increasing UVB exposure time worsened the keratinization, dryness, erythema reaction, and wrinkling of the skin. On the other hand, the skin surface in tricin- or ETZL (50 mg/kg and 150 mg/kg)-treated mice for 10 weeks showed remarkably reduced erythema and keratinization even after UVB irradiation as compared to the UVB control group. However, in the 300 mg/kg of ETZL-treated group and retinoic-acid-applied group, although the number of coarse wrinkles was lower than that of the normal group, we observed the presence of hyperkeratosis, an erythema phenomenon, and rough wrinkles. Next, skin replicas were collected and observed by the SILFLO casting method to compare the degrees of wrinkle formation in each group at 1, 6, 8, and 10 weeks. In the UVB control group, we have indicated the formation of deep coarse wrinkles, which were markedly reduced in the tricin- and ETZL (150 mg/kg)-treated hairless mice groups during the 14-week oral administration as shown in Figure 2B. In the ETZL (300 mg/kg) group, deep wrinkles were formed under UVB irradiation for 10 weeks, which was consistent with the visual assessment results; also, the number of wrinkles was smaller than that observed in the UVB-irradiated group. On the other hand, in the retinoic acid (0.05%)-applied group, there were relatively less wrinkles compared to the UVB control group until 8 weeks of skin irradiation, but increased deep wrinkles were observed at 10 weeks in the skin replica. Considering the above results, we suggest that oral administration of tricin or ETZL (50 and 150 mg/kg) to hairless mice most effectively protects the epidermal keratinocytes and thus prevents wrinkling of skin after chronic UVB exposure.

### 2.4. Effects on Changes in the Moisture Content of the Dorsal Skin

Moisture contents of the dorsal skin were monitored at 5 to 10 weeks, and the contents at week 10 are presented in Figure 2C. The moisture content of the dorsal skin was significantly decreased in the UVB-irradiated control group compared with the non-UVB normal group (*p* < 0.001). The moisture contents of the tricin-administered group and all ETZL-administered groups showed a significant increase as compared to the control group (*p* < 0.05), although the effect in the ETZL group was not dose-dependent. Also, no significant differences in the moisture content were observed between orally administrated ETZL and topically administrated retinoic acid, a reference molecule. These results indicate that tricin or ETZL administration prevents the decrease in dorsal skin moisture content induced by repeated exposure to UVB.

### 2.5. Effects on Histological Difference of the Dosal Skin

**Figure 3:**
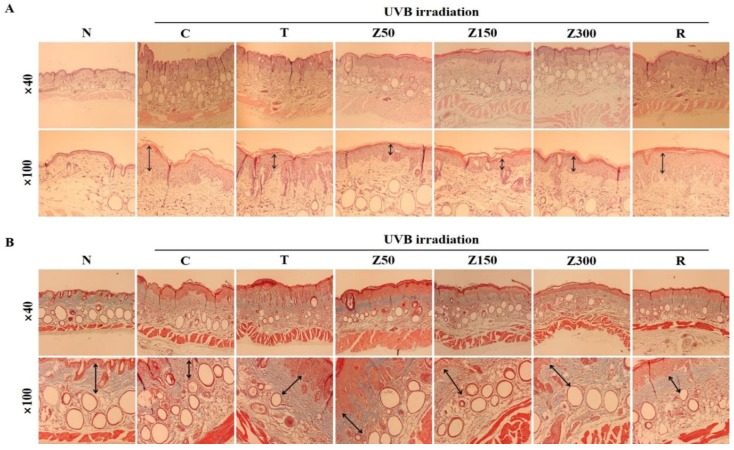
Histological analysis of dorsal surface in tricin- or ETZL-administered SKH-1 hairless mice exposed to UVB irradiation. (**A**) Dorsal skin sections stained with H&E; (**B**) Masson’s trichrome staining. Histological changes were observed at x40 (upper panels) and x100 (lower panels). N, normal control; C, UVB-irradiated control; T, tricin (0.3 mg/kg) oral administration with UVB irradiation; Z50, ETZL (50 mg/kg) oral administration with UVB irradiation; Z150, ETZL (150 mg/kg) oral administration with UVB irradiation; Z300, ETZL (300 mg/kg) oral administration with UVB irradiation; R, UVB irradiation with dermal application of 0.05% retinoic acid.

The dorsal skin sections from SKH-1 hairless mice in each group were stained with hematoxylin and eosin (H&E) or Masson’s trichrome to determine the histological changes in the dermal layer and collagen fibers, respectively. Significant epidermal hyperplasia and degenerated elastic fibers are used as indicators for wrinkle formation and skin photoaging [20,21]. Repeated UV exposure causes collagen fiber degradation in skin tissue, leading to the formation of coarse wrinkles [22]. H&E staining revealed that the epidermal thickness and number of wrinkles in the week 10 UVB-irradiated group was obviously increased compared with the normal group, and tricin or ETZL (50 and 150 mg/kg) exposure significantly decreased the epidermal thickness and deep wrinkles (Figure 3A). The retinoic-acid-treated group also showed a mild effect in preventing wrinkle formation.

On the other hand, Masson’s staining revealed that the collagen fibers were destructed after UVB irradiation, but tricin or ETZL treatments protected the collagen fibers against the UVB-induced degradation, thus preventing wrinkle formation (Figure 3B). Especially, a lower dose of ETZL showed stronger blue staining as compared to the normal group. These results show that ETZL (50 and 150 mg/kg) administration provides better preventive effects against wrinkling and skin damage than a high dose of ETZL (300 mg/kg) or tricin alone, even though the histological changes were significantly prevented in the tricin-treated group and all ETZL-treated groups as compared to the UVB control group.

Taken together, these results indicate that oral administration of ETZL for 14 weeks ameliorates photo-damage, including rough skin surface, dryness, keratinization, and wrinkling in chronic UVB-irradiated hairless mice.

### 2.6. Effects on Collagen and MMPs Proteins Expression

To determine whether the administration of tricin or ETZL to UVB-irradiated hairless mice affects collagen synthesis and degradation, the protein expressions of collagen I as well as MMP-1 (collagenase 1) and -13 (collagenase 3) were measured in mice skin tissue of each group. UV irradiation induces the expression of MMPs in normal human epidermis, resulting in the reduction of collagen fibers, thereby causing wrinkles [23]. Also, excessive exposure of the skin to UV results in excessive production of ROS, which act on keratinocytes to inhibit collagen synthesis in the dermal fibroblasts, thereby promoting the degradative enzyme activity to form wrinkles [24,25].

**Figure 4:**
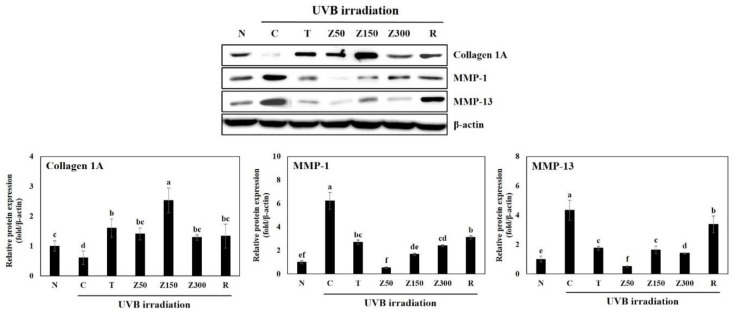
Effects of tricin or ETZL administration on metalloproteinase (MMP)-1, -13, and collagen I protein expression in UVB-irradiated hairless mice skin tissues. Skin tissues were homogenized and the lysates were subjected to Western blotting. Each bar represents the quantitative analysis of proteins expression normalized by β-actin. N, normal control; C, UVB-irradiated control; T, tricin (0.3 mg/kg) oral administration with UVB irradiation; Z50, ETZL (50 mg/kg) oral administration with UVB irradiation; Z150, ETZL (150 mg/kg) oral administration with UVB irradiation; Z300, ETZL (300 mg/kg) oral administration with UVB irradiation; R, UVB irradiation with dermal application of 0.05% retinoic acid. Values are expressed as mean ± SD of three replicates. Different letters show a significant difference at *p* < 0.05 as determined by Duncan’s multiple range test.

Despite UVB irradiation, the level of collagen I expression was increased in all groups with tricin or ETZL oral administration compared to the control group (Figure 4). Especially, the ETZL (150 mg/kg) group showed a marked increase in collagen I expression (*p* < 0.001), while a minimal effect was observed in the ETZL (50 mg/kg) group. We also observed an increasing trend in tricin or retinoic acid treatment. In addition, the expression of MMP-1 and MMP-13, which are known as collagen-degrading enzymes, was increased in the UVB-control group, whereas the expression levels of MMP-1 were significantly reduced in the tricin-administered group and the ETZL (50 and 150 mg/kg)-administered groups. The expression levels of MMP-13 were also remarkably decreased at all doses of ETZL as well as tricin treatment. However, MMP-1 and MMP-13 expression in the retinoic-acid-applied group increased more than that of the control group, indicating that the application of retinoic acid three times a week did not affect the collagen synthesis and degradation mechanism. As a result, excessive expressions of collagenase, MMP-1, and MMP-13, and decreased collagen I expression, were observed by the UVB-irradiated control. Tricin or ETZL (50 and 150 mg/kg) treatment resulted in an increase in the collagen I expression and a decrease in the MMP-1 and MMP-13 expression, which is thought to help suppress the formation of wrinkles [26,27,28]. These in vivo observations were consistent with the previous in vitro study that found that tricin and ETZL treatments in HDFs result in a significant suppression of MMP (1, 2, 3, 9, 13) release and an enhancement of type-1 procollagen production in UVB-irradiated HDFs, possibly through inhibition of the phosphorylation of MAPKs, such as Akt, JNK, ERK, and p38, thereby downregulating the activities of NF-kB and AP-1. Therefore, it is possible that the inhibition of MMP expression and collagen degradation by tricin or ETZL administration to hairless mice may prevent wrinkle formation in UVB-irradiated mice via NF-kB/AP-1 signaling.

Collectively, our study provides the first evidence that ETZL contains high amounts of tricin that may contribute to the prevention of skin photoaging by UVB irradiation in SKH-1 hairless mice. Notably, 50 mg/kg and 150 mg/kg administration of ETZL exhibit better effects than 300 mg/kg ETZL or tricin alone, suggesting that oral administration of 150 mg/kg ETZL may be the peak effective dose. In addition, 0.3 mg/kg administration of tricin was equivalent to approximately 300 mg/kg ETZL, and tricin-treated mice showed similar ameliorative effects, indicating that tricin may be the dominant active component for protection from UVB-induced skin damage in hairless mice. In conclusion, oral administration of tricin-containing ETZL to UVB-exposed hairless mice could protect the skin against photoaging by maintaining levels of collagen and moisture content and by preventing histological destruction through MMP expression. Overall, our results suggest that tricin as well as *Z. latifolia* are potential materials for anti-wrinkle dietary supplements.

## 3. Materials and Methods

### 3.1. Plant Material and Preparation of ETZL

The aerial parts of *Z. latifolia* were purchased from the Pureunsan Agricultural Association Corporation (Dongdaemun-gu, Seoul, Korea) and ETZL was provided by the BTC Corporation (Sangnok-gu, Ansan, Korea). Briefly, the dried leaves of *Z. latifolia* were incubated with a mixed hydrolysis enzyme (cellulase, hemicellulase, and pectinase) at 35 °C for 16 h in H_2_O, and enzymes were inactivated with heat. The extracted solution was filtered and acquired. After the extraction of the residual with 70% EtOH (Duksan Science, Seoul, Korea) at 80 °C for 6 h, the filtered and extracted solution was mixed with the first enzyme extract, and the total enzyme extract was concentrated and dried to produce ETZL.

### 3.2. HPLC Analysis

HPLC was performed with an Agilent Infinity 1260 (Agilent Technologies, Palo Alto, CA, USA). The column was a SUPELCO Discovery^®^ C18 (4.6 × 250 mm, 5 µm) (Sulpelco, Bellefonte, PA, USA) and the separation of tricin was operated at 30 °C using an Agilent 1260 Infinity Diode Array Detector. The mobile phase composition was A: 0.15% phosphoric acid in H_2_O B; methanol with a flow rate of 1.0 mL/min. The gradient elution conditions were as follows: 0–3 min, 20% B; 3–8 min, 20–50% B; 8–20 min, 50–55% B; 20–30 min, 55–85% B; 30–45 min, 85–20% B.

### 3.3. Experimental Animals and UV Irradiation

Six-week-old female SKH-1 hairless mice were obtained from Orient Bio (Seongnam, Korea) and housed at the Southeast Medi-Chem Institute (SEMI; accredited unit no. 412). The animals were housed at 26 ± 1 °C and 50 ± 5% relative humidity under a 12-h light–dark cycle. All animals were fed AIN-93G (ALTROMIN, Lage, Germany) and fresh water ad libitum. After an acclimation for 7 days, mice were randomly divided into seven groups of nine animals: normal control (−UVB, N), UVB-irradiated control (+UVB, C), positive control (UVB + retinoic acid 0.05%, skin, R), tricin 0.3 mg/kg (UVB + tricin 0.3 mg/kg, p.o., T), ETZL 50 mg/kg (UVB + ETZL 50 mg/k, p.o., Z50), ETZL 150 mg/kg (UVB + ETZL 150 mg/k, p.o., Z150), and ETZL 300 mg/kg (UVB + ETZL 300 mg/k, p.o., Z300). ETZL and tricin were orally administered to the animal in each group daily for 14 weeks, while retinoic acid was administered onto skin in the positive control group three times a week for 14 weeks. The normal and control groups were orally administered saline. Food intake and body weight were regularly measured throughout the study. UVB irradiation was performed for 10 weeks. The mice were sacrificed 4 days after the final irradiation for recovery from the acute UVB exposure. For animal euthanasia, abdominal aorta was cut after CO_2_ anesthesia, organs were examined visually, and organs were weighed. The animal protocol used in this study was reviewed and approved by the SEMI Institutional Animal Care and Use Committee (SEMI-16-13).

The animals were kept in a stainless cage (JEUNGDO BIO&PLANT, Seoul, Korea) and subjected to UVB irradiation (302 nm, 0.3 mW/cm^2^) by an array UVB lamp (UV-1000; DongSeo Science Co., Ltd., Seoul, Korea). UV energy was measured with a UV radiometer. The mice were irradiated three times a week starting with 60 mJ/m^2^, and the minimal erythemal dose (MED) was set at 60 mJ/m^2^. The intensity of irradiation was gradually increased by 1 MED per week during the first 4 weeks, and the mice were irradiated at 4 MED of UVB from week 5 to 10. The non-irradiated normal group was treated identically without UVB exposure.

### 3.4. Serum Biochemical Analysis

Before blood collection via abdominal vein, all the mice were fasted for 15 h, and the serum was obtained by centrifuging the blood after incubation for 30 min at room temperature. The hepatotoxicity of tricin and ETZL was assessed by measuring AST and ALT in serum using an automated serum analyzer (COBAS C111, Roche Diagnostics, Basel, Switzerland).

### 3.5. Morphological and Histological Observation

Morphologic changes of mouse dorsal skin exposed to UVB irradiation were observed at 1, 6, 8, and 10 weeks using a digital microscope (NIKON, Tokyo, Japan), and the dorsal skin surface was replicated using SILFLO (Flexico development LTD., Tokyo, Japan). For histological assessment, the dorsal skin was collected and fixed with 10% formalin for 24 h, dehydrated in ethanol, and then embedded in paraffin wax. The 5-µm-thick skin sections were stained with hematoxylin and eosin (H&E), and the 4 µm-thick sections were stained using a Masson’s trichrome staining kit (American Tech Scientific; Lodi, CA, USA). The images of stained slides were taken using a light microscope (Nikon, Tokyo, Japan).

### 3.6. Measurement of Skin Moisture

Mice were maintained in a room at 26 ± 3 °C with a relative humidity of 50 ± 4% for 14 weeks. Skin moisture was measured once a week from 5 to 10 weeks with a Corneometer CM 825 (Courage-Khazake, Koln, Germany).

### 3.7. Western Blot

The mice dorsal skin (20 mg) was homogenized in lysis buffer (50 mM Tris-HCl, 150 mM NaCl, 5 mM EDTA, pH 8.0), the prepared lysates were centrifuged at 14,000 rpm for 20 min at 4 °C, and equal amounts of total proteins were separated by 10% SDS-PAGE and transferred to polyvinylidene fluoride (PVDF) membranes. The membranes were blocked with 5% non-fat milk in TBS containing 0.1% Tween 20 (TBS-T). The membranes were then incubated with primary antibodies (anti-collagen I, anti-MMP-1, anti-MMP-13) at 4 °C overnight in 5% non-fat milk in TBS-T and washed with TBS-T. Subsequently, the membranes were incubated with horseradish peroxidase (HRP)-conjugated secondary antibodies (goat anti-rabbit IgG or goat anti-mouse IgG Ab, diluted 1:2000, Thermo Fisher, Waltham, MA, USA). Beta actin (SantaCruz, CA, USA) was used as a loading control for all proteins. Quantification of proteins was performed using an enhanced chemiluminescence Western Blot detection kit (Abfrontier, WEST SAVE GOLD, Seoul, Korea).

### 3.8. Statistical Analysis

Data were presented as means ± SD values of three experiments. All data were analyzed by one-way ANOVA using IBM SPSS version 20 (SPSS, Chicago, IL, USA). Differences among groups were examined using Duncan’s multiple range test and a significant difference was considered at *p* < 0.05.

## Figures and Tables

**Table 1:** Tricin contents of *Zizania latifolia* (ETZL) depend on enzyme treatment time.

Enzyme Treatment Time (h)	Extraction Yield (%)	Tricin Content (mg/100 g) *
0	10.74	19.7
4	16.54	16.5
8	16.92	19.3
12	17.36	21.2
16	17.45	25.0
24	15.72	24.2

* Total tricin content in dried *Z. latifolia* (100 g).
